# Respiratory Syncytial Virus (RSV) Hospitalization Seasonal Patterns and Economic Burden in the US: Implications for Further Optimizing the Use of RSV Preventives

**DOI:** 10.3390/vaccines13040366

**Published:** 2025-03-29

**Authors:** Amy W. Law, Jay Lin, Jennifer Judy, Sarah J. Pugh, Alejandro Cane

**Affiliations:** 1 Global Access & Value, Pfizer, Inc., New York, NY 10001, USA; 2 Outcomes Research, Novosys Health LLC, Miami, FL 33145, USA; jay.lin@novosyshealth.com; 3 Global Respiratory Vaccines, Real World Evidence, Pfizer, Inc., New York, NY 10001, USA; jennifer.judy@pfizer.com; 4 Vaccines, US Medical and Scientific Affairs, Pfizer, Inc., Collegeville, PA 19426, USA; sarah.pugh@pfizer.com (S.J.P.); alejandro.cane@pfizer.com (A.C.)

**Keywords:** respiratory syncytial virus, hospitalization, infant, seasonal variation, off-season

## Abstract

Background/Objectives: The CDC has recommended immunizations to protect infants during the respiratory syncytial virus (RSV) season, which varies annually and geographically. Seasonal differences in RSV hospitalizations among infants are not well studied. Methods: This retrospective cohort study identified infants < 12 months old hospitalized with RSV from the PINC AI Healthcare Database during the 2018–2023 surveillance years (1 July–30 June). Monthly RSV hospitalizations were stratified by U.S. census division and age group (<3, 3–5, 6–8, 9–11 months). Patient characteristics, healthcare resource utilization (HCRU), and cost were compared between typical in-season months (October–March) and typical off-season months (April–September) for RSV hospitalizations. Results: Among 20,531 hospitalizations for RSV (mean age: 4.1 months, 56.4% male), 22% (*n* = 4510) were off-season; 83% occurred in June–September across US census divisions. Infants < 3 months accounted for 46% (*n* = 2054) of off-season hospitalizations. Seasonal patterns were similar across age groups. Off-season hospitalizations were associated with longer hospital length of stay (6.9 vs. 4.9 days) and more supplemental oxygen (59.1% vs. 55.5%), intensive care unit admission (30.1% vs. 26.8%), and mechanical ventilation/airflow usage (20.3% vs. 16.3%). Mean hospitalization costs were 40% higher during off-season ($17,911 vs. $12,757). In the surveillance years before (2018–2020) and after (2022–2023) the COVID-19 pandemic, off-season costs and HCRU were consistently higher than in-season. Conclusions: There is an unmet need among the 1 in 5 infants with off-season RSV hospitalizations, which are associated with higher HCRU and costs. Current recommendations on RSV preventives offer limited protection for infants exposed to RSV outside the typical season.

## 1. Introduction

Respiratory syncytial virus (RSV) is the primary cause of infant lower respiratory tract illness. The most common cause of inpatient admission among United States (US) children under the age of 2 years, the mean annual RSV hospitalization rate among infants < 1 year of age has been estimated to be 20 per 1000 [1,2]. More than 50% of RSV hospitalizations occur within the first 3 months of life and over 75% occur by 6 months of age [3].

In 2023, two new immunization products were licensed and recommended for the prevention of RSV disease among infants, though most infants do not need both. A maternal RSV prefusion F protein-based vaccine (RSVpreF) administered to pregnant persons 32 through 36 weeks’ gestation prevents RSV disease in infants aged through 6 months [4]. Alternatively, nirsevimab, a long-acting monoclonal antibody, is administered to infants aged <8 months who are born during or entering their first RSV season and lasts through at least 5 months [5].

The US Centers for Disease Control and Prevention (CDC) Advisory Committee on Immunization Practices (ACIP) recommended seasonal administration of each prevention option timed to the typical national onset and offset of the RSV season. Surveillance data informed the recommendation according to the timing of high transmission rates occurring throughout the year. Maternal RSVpreF vaccination is recommended from September through January and nirsevimab is recommended from October through March [4,5,6,7,8]. While the timing of these seasonal recommendations is for most of the continental US, annual and geographic variability in RSV seasonality exists [8,9,10].

However, it remains unclear how the temporal and geographic patterns of RSV seasonality may vary among infants < 1 year old and whether any inequities exist according to the timing of birth out of season [6,8,11,12]. Given the current recommendations for seasonal administration of infant RSV preventions, understanding the clinical and economic burden of RSV hospitalizations among infants < 1 year occurring off-season is of importance. This study aimed to characterize and assess the seasonal patterns of hospitalization due to RSV among infants < 12 months of age. In addition, the economic burden of RSV hospitalizations occurring during the RSV season (i.e., 1 October–31 March) and off-season (i.e., 1 April–30 September) was investigated.

## 2. Materials and Methods

### 2.1. Study Design and Data Source

This descriptive retrospective cohort study identified infants < 12 months of age hospitalized with RSV using the PINC AI Healthcare Database (PHD). The PHD is a hospital-based data source with >1190 contributing hospitals representing approximately 25% of annual hospital admissions and 326 million unique patients across the US. The regional and rural–urban distribution of PHD hospitals is similar to American Hospital Association member hospitals [13]. All data are de-identified and fully compliant with the Health Insurance Portability and Accountability Act (HIPAA).

### 2.2. Study Population

Infants < 1 year of age hospitalized with RSV during July 2018–June 2023 were identified by a primary or secondary discharge diagnosis or a positive RSV lab test result. International Classification of Diseases, Tenth Revision, Clinical Modification (ICD-10-CM) diagnosis codes for RSV included B97.4, J12.1, J21.0, and J20.5. Hospital lab test records were also searched to identify positive RSV lab tests. To assess trends in RSV hospitalization by infant age in months, RSV patients were required to have a “newborn” record, defined as a diagnosis code for liveborn infants according to place of birth (ICD-10-CM: Z38.00–Z38.8), since the PHD’s default patient demographic information only reports age by year. The infant’s age in months was estimated by calculating the number of months from the “newborn” record to the RSV hospitalization record. All qualified RSV hospitalizations for infants < 12 months of age were included in the study.

### 2.3. Outcomes

Patient demographic, hospital, and clinical characteristics were evaluated during the RSV hospitalization. Chronic medical conditions were identified by ICD-10-CM diagnosis codes present during RSV hospitalizations. Healthcare resource utilization (HCRU) and costs evaluated included total inpatient cost, hospitalization length of stay (LOS), inpatient pharmacy usage and costs, as well as the frequency, duration, and costs of intensive care unit (ICU) admission, mechanical ventilation/airflow (MVAF) usage, and supplemental oxygen usage. ICU admissions were identified by hospital chargemaster, Healthcare Common Procedure Coding System (HCPCS) and Current Procedural Terminology, fourth edition (CPT-4) procedure codes; MVAF was identified by hospital chargemaster, ICD-10 procedure codes (ICD-10-PCS), HCPCS, and CPT-4 codes; and supplemental oxygen was identified by hospital chargemaster and ICD-10-PCS. The duration of healthcare resource usage was measured based on the distinct number of days with a recorded corresponding service day and a relevant standard charge code or code during the inpatient encounter. Costs were assessed among patients with available cost data for the service category. All costs are inflation adjusted to 2024 US dollars using Consumer Price Index Medical Care component data.

### 2.4. Data Analyses

RSV hospitalizations were stratified by seasonality, surveillance year, age group, and geographic region for analysis. RSV seasonality was defined according to current ACIP recommendations for the prevention of RSV among infants with admission dates during 1 October–31 March being in-season and those during 1 April–30 September being off-season [5]. Based on admission date, hospitalizations were stratified into 5 surveillance years defined as the period of 1 July–30 June of the following year. Age groups included infants 0–2, 3–5, 6–8, and 9–11 months at hospitalization. Additional evaluations were conducted among infants 0, 1, and 2 months of age. Geographic stratifications were based on hospital location within the 9 US census divisions.

Patient demographic, hospital, and clinical characteristics, as well as HCRU and costs were assessed for the overall study population (infants < 12 months of age) and compared between all in-season and off-season RSV hospitalizations (July 2018–June 2023). HCRU and costs were also evaluated for in-season and off-season hospitalizations during the pre-COVID (July 2018–June 2020) and post-COVID (July 2022–June 2023) periods. HCRU and costs were further stratified among infants born within or outside an RSV season to evaluate the potential burden of a subgroup that is not eligible for any RSV prevention despite the current recommendation on RSV prevention. Infants born within an RSV season (i.e., between 1 October–31 March) were defined as in-season births and those born outside an RSV season were defined as off-season births. The seasonality of RSV cases among all infants < 1 year, regardless of an identifiable age in months, was evaluated as a sensitivity analysis.

Patient and hospital characteristics as well as HCRU and cost outcomes were summarized using descriptive statistics. For categorical variables, the frequency and percentage of patients in each category were presented. Percentages were based on the total number of relevant patients without ‘unknown’ or missing values. For continuous variables, data were presented as means with standard deviations (SD) and medians. Analysis of variance (ANOVA) and Chi-squared tests were used as statistical comparisons for continuous and categorical variables, respectively. All data analyses were performed using statistical software SAS version 9.4 (SAS Institute; Cary, NC, USA).

## 3. Results

### 3.1. Study Population Characteristics

**Figure 1:**
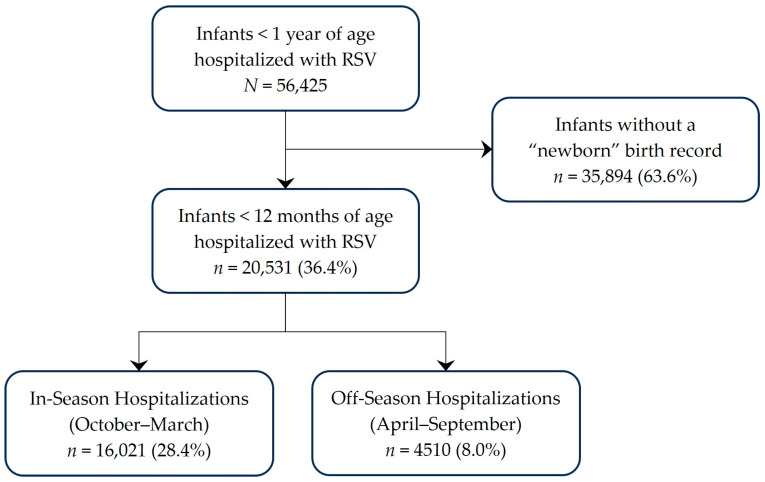
Study Population.

Among infants < 1 year of age hospitalized with RSV during July 2018–June 2023, 36% (*n* = 20,531) were determined to be <12 months of age when hospitalized (Figure 1). Approximately one-fifth of these RSV hospitalizations occurred outside the typical RSV season, which runs from 1 October–31 March. Of these off-season hospitalizations, 2774 (61.5%) occurred during the pandemic surveillance years. The demographic and clinical characteristics of infants < 12 months of age when hospitalized with RSV are reported in Table 1. Although patient age and sex distribution were similar between off-season and in-season hospitalizations, there were some differences in the demographic and clinical profiles of infants who were hospitalized in-season and off-season. A greater proportion of infants hospitalized off-season were Black, hospitalized in the South, and had congenital heart disease or other respiratory conditions during the RSV hospitalization; a smaller proportion were Hispanic, had Medicaid, and had pneumonia while hospitalized with RSV.

### 3.2. Seasonal Distribution of RSV Hospitalizations

**Figure 2:**
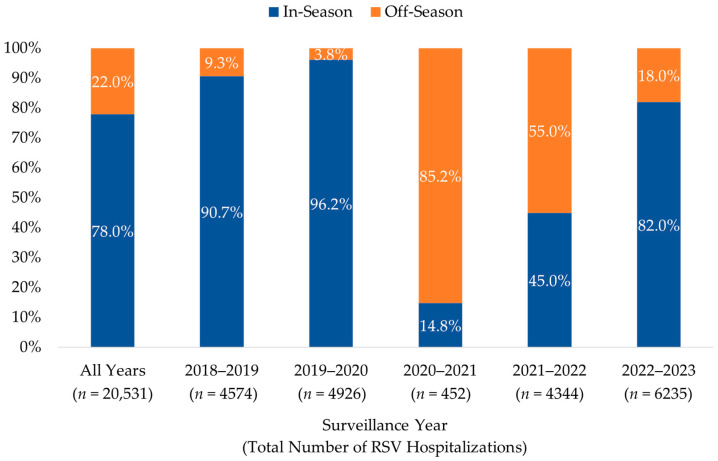
Distribution of RSV Hospitalizations by Surveillance Year among Infants < 12 months, 2018–2023. The percentage of infants with an RSV hospitalization during (in-season, 1 October–31 March) and outside (off-season, 1 April–30 September) a typical RSV season are shown for each surveillance year (1 July to 30 June of the next year).

The distribution of in- and off-season RSV hospitalizations among infants < 12 months of age during each of the five surveillance years is illustrated in Figure 2. The seasonal distribution of RSV hospitalizations was significantly impacted by the COVID-19 pandemic, particularly during the first of the two pandemic surveillance years (July 2020–June 2021), where 85.2% (*n* = 385) of cases occurred during months typically considered to be off-season. Although the number of cases returned to pre-pandemic (2018–2020) levels during the second year, 55.0% (*n* = 2389) of hospitalizations occurred during the off-season months. Post-pandemic RSV hospitalization levels during July 2022–June 2023 were higher than pre-pandemic levels during the July 2018–June 2019 surveillance year, with a greater proportion occurring in the off-season (18.0% vs. 9.3%). Sensitivity analysis showed consistent seasonal patterns among all infants < 1 year of age.

**Figure 3:**
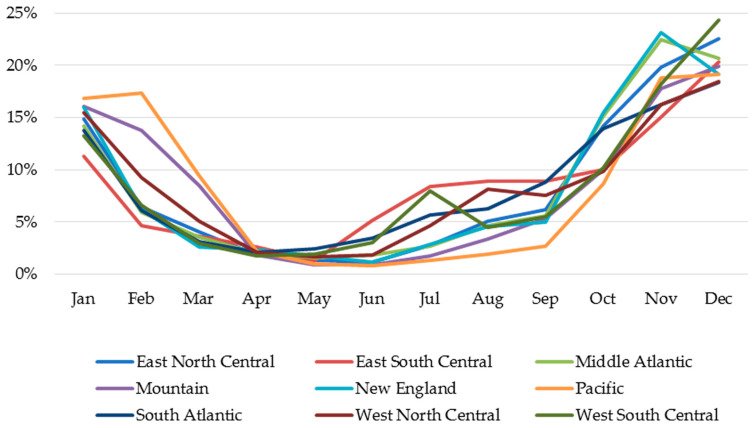
Monthly Distribution of RSV Hospitalizations Stratified by Geographic Region Across All Study Years.

The monthly distribution of RSV hospitalizations among infants < 12 months of age across all surveillance years, stratified by the nine US census divisions, is illustrated in Figure 3. Seasonal distributions varied geographically from 9.9% of cases in the Pacific division to 35.1% of cases in the East South Central division occurring during off-season months. Of the RSV hospitalizations that occurred during the off-season, 83% of these cases occurred in June–September, ranging across various divisions from 67.9% in the Pacific division to 89.0% in the East South Central division.

**Figure 4:**
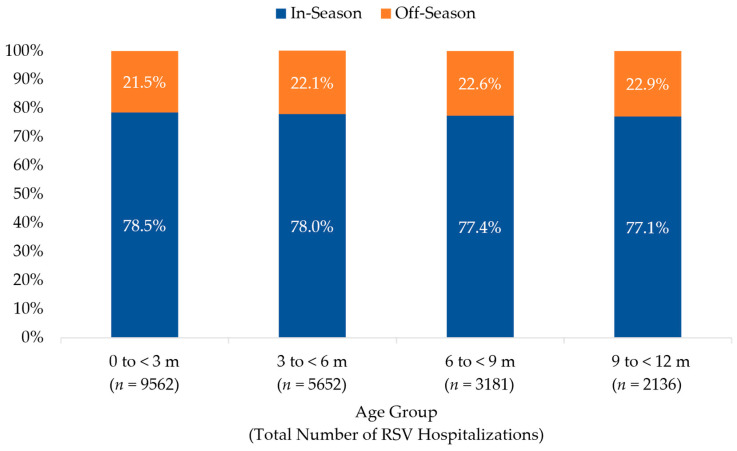
Distribution of RSV Hospitalization by Age Group among Infants Age < 12 months, 2018–2023. The percentage of infants with an RSV hospitalization during (in-season, 1 October–31 March) and outside (off-season, 1 April–30 September) a typical RSV season are shown for each age group (in months).

The distribution of in- and off-season RSV hospitalizations among infants < 12 months of age across all surveillance years, stratified by age group, is illustrated in Figure 4. The seasonality of RSV hospitalizations did not vary by age group. Infants < 3 months of age accounted for the 46.6% (*n* = 9562) of all RSV hospitalizations; 30.6% (*n* = 2923) of these infants were <1 month of age when hospitalized with RSV and 39.2% (*n* = 3746) were 1 to <2 months of age. Of all off-season hospitalizations for RSV, 45.5% (*n* = 2054) were among infants < 3 months of age; 32.1% (*n* = 660) of these infants were <1 month, and 38.1% (*n* = 782) were 1 to <2 months of age when hospitalized.

### 3.3. HCRU and Costs

The HCRU and cost of all RSV hospitalizations during July 2018–June 2023, as well as a comparison of the HCRU and cost of RSV hospitalizations during in-season and off-season months are reported in Table 2. Off-season hospitalizations were associated with significantly longer mean hospital LOS (7 vs. 5 days, *p* < 0.0001), more ICU admissions (30.1% vs. 26.8%, *p* < 0.0001), MVAF use (20.3% vs. 16.3%, *p* < 0.0001), and supplemental oxygen use (59.1% vs. 55.5%, *p* < 0.0001). Furthermore, infants hospitalized during the off-season had longer ICU admissions and required more days with MVAF and supplemental oxygen use. Overall, mean total hospitalization costs were 40% higher during the off-season, primarily driven by ICU usage, which, unlike MVAF and oxygen use, was associated with significantly higher mean costs ($24,311 vs. $18,365, *p* = 0.0003).

**Figure 5:**
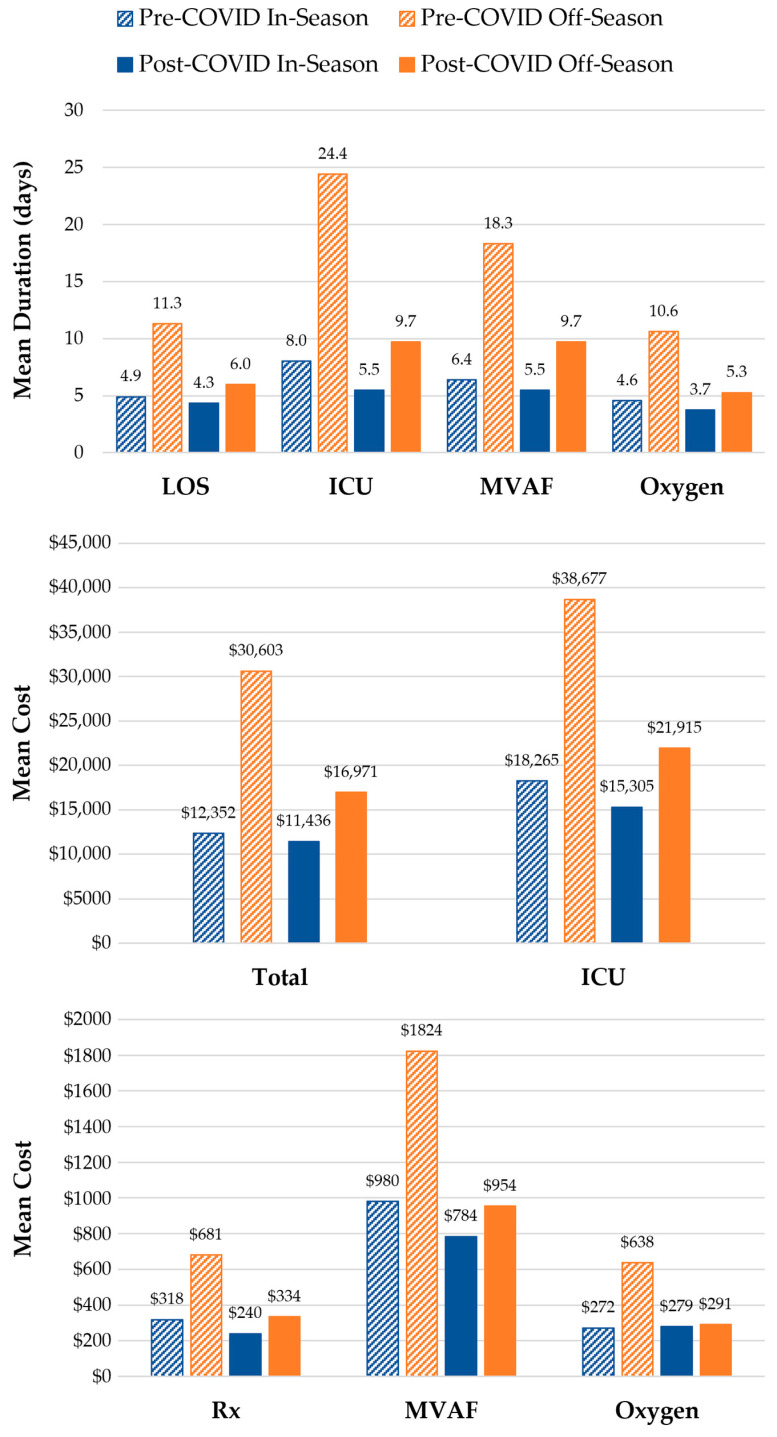
In-Season and Off-Season HCRU and Costs during the Pre-COVID (July 2018–June 2020) and Post-COVID (July 2022–June 2023) Periods. Hospital LOS and total costs were evaluated for all RSV hospitalizations among infants < 12 months of age during (in-season, 1 October–31 March) and outside (off-season, 1 April–30 September) a typical RSV season. The duration of ICU admissions, MVAF, and supplemental oxygen usage were assessed among infants who received such services while hospitalized. Service costs were assessed among the subset of those who with available cost data. Pre-COVID in-season vs. off-season comparison: *p* < 0.0001 for all HCRU and costs except for the cost of MVAF (*p* = 0.003). Post-COVID in-season vs. off-season comparison: *p* < 0.0001 for all HCRU except for MVAF duration (*p* = 0.0003); *p* = 0.02 for Rx and ICU costs; *p* = 0.32 for MVAF costs; and *p* = 0.69 for supplemental oxygen costs.

Comparisons of the HCRU and costs of in-season and off-season RSV hospitalizations during the pre-COVID (July 2018–June 2020) and post-COVID (July 2022–June 2023) periods are illustrated in Figure 5. In the two surveillance years prior to the COVID-19 pandemic (2018–2020), 9500 infants were hospitalized with RSV; 6.5% (*n* = 615) of these hospitalizations were during off-season months. Of all five surveillance years, the last post-pandemic year (2022–2023) had the highest number of cases (*n* = 6235), of which 18.0% (*n* = 1121) occurred during the off-season. The HCRU and costs of off-season RSV hospitalizations were consistently higher than in-season hospitalizations both prior to and after the COVID-19 pandemic surveillance years (July 2020–June 2022). Hospital LOS and the duration of ICU admissions, MVAF, and supplemental oxygen use were all longer for off-season hospitalizations during both the pre-COVID (2018–2020) and post-COVID (2022–2023) periods. However, while healthcare costs were higher for most services received during off-season hospitalizations within the pre-COVID (2018–2020) and post-COVID (2022–2023) periods, the cost of MVAF and supplemental oxygen use was comparable between in- and off-season hospitalizations during the post-COVID period.

The HCRU and costs of in-season and off-season RSV hospitalizations among infants born in-season and those born off-season are reported in Table 3. Although similar proportions of infants were born in- and off-season (48.0% vs. 52.0%), off-season RSV hospitalizations were more frequent among infants born off-season vs. in-season (25.6% vs. 18.1%). Mean off-season RSV hospitalization HCRU was 2.4- to 4.0-fold higher and mean costs were 1.7- to 2.9-fold higher among infants born in the RSV off-season compared to infants who were born in-season.

## 4. Discussion

This large-scale, real-world database analysis showed that over one in five infants hospitalized with RSV were affected during the off-season, with rates varying by geography and year. Nearly half of these hospitalizations occurred among infants < 3 months of age; 32.1% of these infants were <1 month when hospitalized. Off-season hospitalizations with RSV were associated with longer hospital stays, higher ICU admission, a greater need for MVAF and supplemental oxygen for longer durations, and higher costs. Post-pandemic RSV hospitalization levels during the 2022–2023 surveillance year were higher than pre-pandemic levels during the 2018–2019 surveillance year, with a greater proportion occurring in the off-season. During both the 2018–2020 pre-pandemic and 2022–2023 post-pandemic surveillance years, off-season hospitalizations were generally associated with higher HCRU and costs than in-season hospitalizations. Compared to infants born in-season, more infants born during the off-season were hospitalized with RSV off-season and had more HCRU and higher costs.

Although the CDC’s analysis of surveillance data during 2017–2023 was not limited to pediatric patients, the reported variability in RSV season duration and geographic variation was consistent with our study’s findings [8]. Among 850 children hospitalized with RSV in Texas during May 2000–September 2006, 5.3% were admitted off-season between May and September [13]. In our study, 22.0% of hospitalizations were off-season between April and September. In contrast, only 2.9% of the 109,185 children < 24 months of age who were hospitalized for RSV during July 2017–November 2022 had an off-season hospitalization when variable RSV season durations were employed [9]. From among those <12 months of age, 54.7% of the off-season RSV hospitalizations occurred among infants < 4 months. A study of regional RSV activity during July 2010–June 2013 reported regional variations in the duration of RSV activity with seasons that typically began between October and January and ended between March and May [14]. Furthermore, 9.8% of pediatric RSV hospitalizations occurred outside of these regional activity seasons. Similar to our study, the frequency of off-season RSV hospitalizations varied by region, ranging from 5.6% to 22.4%.

In our study, 49.6% of the infants hospitalized in-season were born off-season, which was similar to the previously reported rate of 54% [15]. Previous studies have also reported a higher risk of hospitalization with RSV among infants born during a typical RSV season, as well as increased ICU admission rates, especially among preterm infants [15,16,17]. In our study, the proportion of infants born in- and off-season who had ICU admissions was similar (28.1% vs. 26.9%). However, the gestational age of 73.6% of the infants in our study was unknown. With only 23.0% (*n* = 2265) of infants born in-season and 25.2% (*n* = 2690) of infants born off-season identified as preterm, RSV hospitalization by preterm birth status was not evaluated in our study.

Numerous studies have investigated the HCRU and costs associated with pediatric RSV hospitalizations [18,19,20,21,22]. However, few have addressed the impact of RSV seasonality. In the study of children hospitalized with RSV in Texas, a greater proportion of patients with off-season hospitalizations received MVAF (8.9% vs. 3.0%, *p* = 0.05) compared to patients admitted in-season [13]. Another study of children < 24 months of age hospitalized for RSV reported more ICU admissions (38.9% vs. 26.5–31.5%, *p* < 0.001) and MVAF (15.8% vs. 8.4–12.0%, *p* < 0.001) during off-season vs. in-season hospitalizations [9]. Our study has demonstrated that off-season RSV hospitalizations are associated with higher HCRU and costs compared to in-season hospitalizations.

In our study, the pre-pandemic period included both the 2018–2019 and 2019–2020 surveillance years. For much of the 2019–2020 RSV season, monthly RSV-associated hospitalization rates among infants < 1 year of age were higher than those corresponding to the previous season, peaking at 281.8 and 211.5 per 100,000 during January 2020 and 2019, respectively [23]. However, by the start of the COVID-19 pandemic in March 2020, the RSV hospitalization rate had dropped below that of the 2018–2019 season (69.5 vs. 84.1 per 100,000) and was substantially lower during April 2020 (3.1 vs. 34.7 per 100,000), the last month of the RSV Hospitalization Surveillance Network (RSV-NET) surveillance period. A similar trend was seen in our study, where the percentage of RSV hospitalizations at the beginning of the RSV season was greater in 2020 than in 2019, before shifting in February (2020: 14.9%; 2019: 16.0%). As the 2019–2020 surveillance year progressed, the disparities in proportions widened (March: 5.9% vs. 10.1%; April: 0.5% vs. 4.0%; May: 0.1% vs. 1.8%; June: 0.02% vs. 1.1%). Correspondingly, while the number of in-season hospitalizations during the 2019–2020 surveillance year may be slightly lower, the number of off-season hospitalizations would be expected to be substantially reduced. As a result, the economic burden of off-season RSV hospitalizations during the pre-pandemic period may be underestimated. The low levels of RSV activity during the fall and winter of the 2020–2021 surveillance year may be attributed to the mitigation measures implemented at the start of the COVID-19 pandemic. Lockdowns, social distancing, and travel restrictions decreased exposure to RSV, reducing the incidence of infections that could lead to hospitalization. However, reduced exposure to RSV also resulted in decreased immunity, whether due to waning maternal immunity or declining herd immunity. This decreased immunity, as well as the easing of mitigation measures, may have contributed to the spikes in off-season RSV hospitalizations that occurred during the 2020–2022 pandemic surveillance years. Although the COVID-19 pandemic impacted RSV seasonality, the difference between in-season and off-season HCRU and costs persisted. The decrease in the magnitude of the difference during the post-COVID timeframe is likely the consequence of shorter hospital LOS during this period.

The findings of this study should be interpreted in the context of several limitations. First, since hospitalizations that occur outside the PHD catchment network are not recorded, it may not be representative of the entire population of hospitalized RSV patients in the US. However, the hospital-based PHD database captures approximately one in four hospitalizations in the US and includes hospitals across all census divisions [24]. In addition, the regional and urban-rural distribution of its member hospitals is comparable to the member hospitals of the American Hospital Association. Second, this administrative database is subject to potential coding and billing errors, and deidentification of patient records prevents validation of outcomes. In addition, identification of preterm infants was limited to ICD-10 codes, which may not be well-documented in claims data. Due to the limited laboratory clinical data available in the PHD, RSV diagnoses were not laboratory confirmed and cases with other viral illnesses identified as RSV may possibly have been included. Third, only a subpopulation of infants hospitalized with RSV had a “linked” newborn birth record, allowing us to estimate age in months. However, sensitivity analysis found that the distribution of all infants hospitalized due to RSV was comparable to the subpopulation of infants with birth records. Fourth, study outcomes were not adjusted for comorbid conditions that could potentially have increased the risk of off-season hospitalization and increased HCRU. Finally, the seasonal distribution of RSV hospitalizations during the pandemic surveillance years diverged from that of previous years, with significantly fewer cases during July 2020–June 2021. The COVID-19 pandemic also altered healthcare seeking behavior, which may have impacted HCRU and cost outcomes. The seasonal distribution of RSV hospitalizations during the 2022–2023 post-pandemic surveillance year appears to be returning to the pre-pandemic levels seen during the 2018–2019 surveillance year; further monitoring of the post-pandemic seasonality of RSV hospitalizations is needed to better inform and optimize prophylactic measures for infants at risk of RSV infection.

## 5. Conclusions

The current ACIP recommendations on seasonal administration of infant RSV prevention options were informed by historical surveillance data. Our study demonstrates that more than one-fifth of infants < 12 months of age had an off-season hospitalization with RSV. While RSV activity appears to be returning to pre-pandemic levels, these off-season cases continue to be associated with longer length of hospital stay, higher ICU admission, and higher costs. More importantly, infants born off-season who were subsequently hospitalized with RSV are unable to benefit from available preventive measures. Our study findings may have important implications for policy makers in their ongoing assessment of recommendations by informing about both implementation timing and how expansion of the vaccination window can reduce the clinical and economic burden of infant RSV occurring off-season and outside the window for current prevention options.

## Figures and Tables

**Table 1:** Patient Demographic, Clinical, and Hospital Characteristics among Infants < 12 months.

	Overall *n* = 20,531	RSV Season		
		In-Season *n* = 16,021	Off-Season *n* = 4510	*p*-Value ^1^
Patient Demographics				
Age, months				0.1592
Mean (SD)	4.1 (3.1)	4.1 (3.1)	4.2 (3.2)	
Median	3.3	3.2	3.3	
Sex, *n* (%)				0.3255
Female	8956 (43.6%)	7010 (43.8%)	1946 (43.2%)	
Male	11,569 (56.4%)	9005 (56.2%)	2564 (56.9%)	
Unknown/Missing	6 (0.03%)	6 (0.04%)	0 (0.0%)	
Race, *n* (%)				<0.0001
White	12,976 (63.2%)	10,219 (63.8%)	2757 (61.1%)	
Black	2867 (14.0%)	2061 (12.9%)	806 (17.9%)	
Asian	461 (2.3%)	366 (2.3%)	95 (2.1%)	
Other/Missing	4227 (20.6%)	3375 (21.1%)	852 (18.9%)	
Hispanic, *n* (%)				<0.0001
Yes	4215 (20.5%)	3416 (21.3%)	799 (17.7%)	
No	13,266 (64.6%)	10,099 (63.0%)	3167 (70.2%)	
Unknown	3050 (14.9%)	2506 (15.6%)	544 (12.1%)	
Health Insurance, *n* (%)				0.0001
Commercial	6807 (33.2%)	5280 (33.0%)	1527 (33.9%)	
Medicare	30 (0.2%)	20 (0.1%)	10 (0.2%)	
Medicaid	12,523 (61.0%)	9863 (61.6%)	2660 (59.0%)	
Other	1171 (5.7%)	858 (5.4%)	313 (6.9%)	
Hospital Characteristics, *n* (%)				
Urban	18,128 (88.3%)	14,125 (88.2%)	4003 (88.8%)	0.2740
Teaching hospital	12,918 (62.9%)	9937 (62.0%)	2981 (66.1%)	<0.0001
Bed size				<0.0001
<100	744 (3.6%)	600 (3.8%)	144 (3.2%)	
100–199	1742 (8.5%)	1416 (8.8%)	326 (7.2%)	
200–299	2868 (14.0%)	2361 (14.7%)	507 (11.2%)	
300–399	2839 (13.8%)	2233 (13.9%)	606 (13.4%)	
400–499	2780 (13.5%)	2250 (14.0%)	530 (11.8%)	
500+	9558 (46.6%)	7161 (44.7%)	2397 (53.2%)	
Region				<0.0001
Midwest	5013 (24.4%)	3902 (24.4%)	1111 (24.6%)	
Northeast	2785 (13.6%)	2281 (14.2%)	504 (11.2%)	
South	8524 (41.5%)	6132 (38.3%)	2392 (53.0%)	
West	4209 (20.5%)	3706 (23.1%)	503 (11.2%)	
Congestive heart failure	1148 (5.6%)	821 (5.1%)	327 (7.3%)	<0.0001
Pneumonia	2648 (12.9%)	2142 (13.4%)	506 (11.2%)	0.0001
Other respiratory condition	8159 (39.7%)	6259 (39.1%)	1900 (42.1%)	0.0002
Diagnosis type				0.4267
Primary diagnosis	13,864 (67.5%)	10,783 (67.3%)	3081 (68.3%)	
Secondary diagnosis	6656 (32.4%)	5229 (32.6%)	1427 (31.6%)	
Positive RSV test ^2^	11 (0.1%)	9 (0.1%)	2 (0.0%)	
RSV infection at birth	392 (1.9%)	246 (1.5%)	146 (3.2%)	<0.0001

^1^ Comparison between in-season (1 October–31 March) and off-season (1 April–30 September) RSV hospitalizations that occurred between 1 July 2018 and 30 June 2023. ^2^ Infants with a positive RSV test, but no primary or secondary diagnoses of RSV.

**Table 2:** In-Season and Off-Season HCRU and Costs.

	Overall *n* = 20,531	RSV Season		
		In-Season *n* = 16,021	Off-Season *n* = 4510	*p*-Value ^1^
LOS (days)				
Mean (SD)	5.3 (13.5)	4.9 (11.2)	6.9 (19.5)	<0.0001
Median	3	3	3	
LOS ranges (days)				0.0399
1–2 days	8912 (43.4%)	6970 (43.5%)	1942 (43.1%)	
3–4 days	6166 (30.0%)	4834 (30.2%)	1332 (29.5%)	
5–6 days	2540 (12.4%)	2002 (12.5%)	538 (11.9%)	
7+ days	2913 (14.2%)	2215 (13.8%)	698 (15.5%)	
Total Cost				<0.0001
Mean (SD)	$13,889 ($45,896)	$12,757 ($38,696)	$17,911 ($65,193)	
Median	$5560	$5578	$5490	
Rx Usage, *n* (%)	19,180 (93.4%)	14,970 (93.4%)	4210 (93.3%)	
Cost				0.0847
Mean (SD)	$346 ($2000)	$333 ($2053)	$393 ($1801)	
Median	$42	$41	$43	
ICU Usage, *n* (%)	5641 (27.5%)	4285 (26.8%)	1356 (30.1%)	<0.0001
Duration (days)				<0.0001
Mean (SD)	9.0 (22.7)	7.7 (18.8)	13.0 (31.6)	
Median	3	3	3	
Cost				0.0003
# w/cost data	5341	4085	1256	
Mean (SD)	$19,763 ($51,279)	$18,365 ($41,296)	$24,311 ($74,914)	
Median	$7824	$8065	$7313	
MVAF Usage, *n* (%)	3520 (17.1%)	2606 (16.3%)	914 (20.3%)	<0.0001
Duration (days)				<0.0001
Mean (SD)	7.5 (19.7)	6.4 (17.0)	10.6 (25.7)	
Median	3	3	3	
Cost				0.1868
# w/cost data	2530	1889	641	
Mean (SD)	$982 ($2207)	$949 ($2072)	$1082 ($2564)	
Median	$438	$443	$381	
Oxygen Usage, *n* (%)	11,560 (56.3%)	8894 (55.5%)	2666 (59.1%)	<0.0001
Duration (days)				<0.0001
Mean (SD)	4.9 (11.7)	4.5 (10.0)	6.1 (16.0)	
Median	3	3	3	
Cost				0.8595
# w/cost data	10,523	8167	2356	
Mean (SD)	$320 ($1885)	$321 ($2088)	$313 ($877)	
Median	$134	$137	$122	

^1^ Comparison between in-season (1 October–31 March) and off-season (1 April–30 September) RSV hospitalizations that occurred between 1 July 2018 and 30 June 2023.

**Table 3:** HCRU and Cost of In-Season and Off-Season RSV Hospitalizations among Infants Born Within or Outside an RSV Season.

	Born In-Season			Born Off-Season	
	In-Season *n* = 8081	Off-Season *n* = 1784		In-Season *n* = 7940	Off-Season *n* = 2726
LOS (days)					
*n*	8081	1784		7940	2726
Mean (SD)	5.9 (14.9)	3.7 (5.5)		3.8 (4.9)	8.9 (24.5)
Median	3	3		3	3
Total Cost					
*n*	8081	1784		7940	2726
Mean (SD)	$15,274 ($48,071)	$9854 ($28,658)		$10,194 ($25,626)	$23,183 ($80,155)
Median	$5774	$5000		$5421	$5810
Rx Cost					
*n*	7419	1711		7551	2499
Mean (SD)	$404 ($2464)	$225 ($878)		$262 ($1543)	$507 ($2214)
Median	$44	$42		$39	$45
ICU					
Duration (days)					
*n*	2292	478		1992	878
Mean (SD)	10.4 (24.5)	4.4 (8.7)		4.6 (7.0)	17.7 (37.9)
Median	3	3		3	4
Cost					
*n*	2183	434		1902	822
Mean (SD)	$22,704 ($50,314)	$10,986 ($27,565)		$13,384 ($26,677)	$31,347 ($89,636)
Median	$8807	$5741		$7490	$8397
MVAF					
Duration (days)					
*n*	1424	286		1177	627
Mean (SD)	8.5 (21.9)	3.9 (6.0)		3.9 (7.1)	13.6 (30.2)
Median	3	2		2	3
Cost					
*n*	1007	226		882	415
Mean (SD)	$1174 ($2623)	$556 ($670)		$692 ($1105)	$1368 ($3112)
Median	$443	$304		$443	$439
Duration (days)					
*n*	4349	1053		4499	1602
Mean (SD)	5.4 (13.7)	3.3 (3.6)		3.6 (3.8)	8.0 (20.1)
Median	3	3		3	3
Cost					
*n*	4034	954		4133	1402
Mean (SD)	$386 ($2851)	$222 ($443)		$259 ($820)	$376 ($1072)
Median	$143	$106		$132	$129

## Data Availability

The datasets presented in this article are not readily available because the study used data that was acquired under a licensing agreement. Requests to access the datasets should be directed to the corresponding author.

## Abbreviations

The following abbreviations are used in this manuscript:

ACIP	Advisory Committee on Immunization Practices
ANOVA	Analysis of variance
CDC	Centers for Disease Control and Prevention
CPT-4	Current Procedural Terminology, fourth edition
HCPCS	Healthcare Common Procedure Coding System
HCRU	Healthcare resource utilization
HIPAA	Health Insurance Portability and Accountability Act
ICD-10-CM	International Classification of Diseases, tenth revision, clinical modification
ICD-10-PCS	International Classification of Diseases, tenth revision, procedure coding system
ICU	Intensive care unit
LOS	Length of stay
MVAF	Mechanical ventilation/airflow
PHD	PINC AI Healthcare Database
RSV	Respiratory syncytial virus
RSV-NET	RSV Hospitalization Surveillance Network
RSVpreF	RSV prefusion F protein-based vaccine
SD	Standard deviation
US	United States
